# P-1348. Acquired Carbapenemase-Producing Gram-Negative Organisms (CPOs) Across the Pond: Surveillance of Antibiotic Resistance in CPOs in England from 2020-2023

**DOI:** 10.1093/ofid/ofae631.1525

**Published:** 2025-01-29

**Authors:** Katherine L Henderson, Rebecca L Guy, Jacquelyn McCormick, Bharat Patel, Dakshika Jeyaratnam, Mariyam Mirfenderesky, Katie L Hopkins, Russell Hope

**Affiliations:** UKHSA, London, England, United Kingdom; UK Health Security Agency, London, England, United Kingdom; UKHSA, London, England, United Kingdom; UKHSA, London, England, United Kingdom; UKHSA, London, England, United Kingdom; UKHSA, London, England, United Kingdom; UKHSA, London, England, United Kingdom; UKHSA, London, England, United Kingdom

## Abstract

**Background:**

Acquired carbapenemase-producing Gram-negative organisms (CPOs) threaten viable antibiotic treatment options. This is the first national look at CPO phenotypic antimicrobial resistance (AMR) rates since becoming statutorily notifiable in England on 01/10/2020.

**Table 1.**
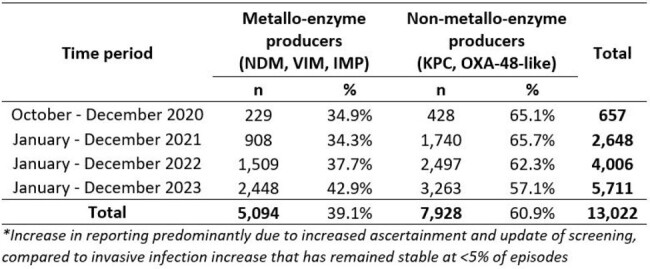
Summary of all big-5 CPO isolates reported from October 2020 to December 2023

**Methods:**

All acquired big-5 (NDM, KPC, OXA-48-like, VIM, IMP) CPO reported specimens and antibiotic susceptibilities were extracted from national laboratory surveillance databases between Oct-2020 to Dec-2023. Records were deduplicated by patient, specimen group (invasive/screening/other), bacterial species and CPO mechanism per year. Regional big-5 CPO population rates were calculated; 2023 AMR data were assessed by mechanism and species.

**Figure 1.**
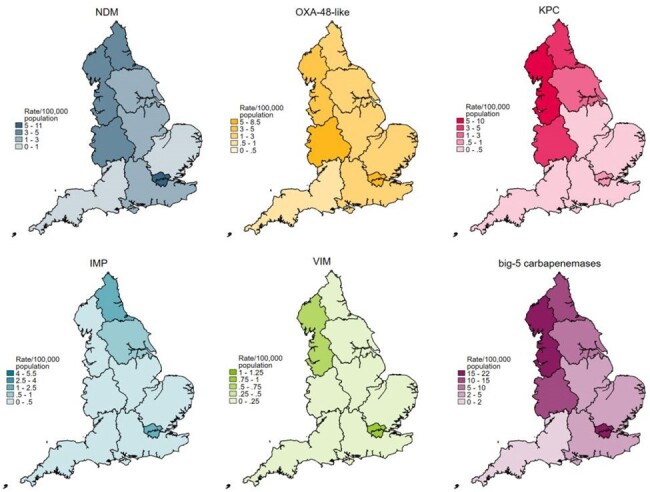
Regional notifications per 100,000 population of acquired CPOs by big-5 carbapenemase mechanisms in England, 2023

**Results:**

In total, 13,022 episodes from 9,945 patients were reported; median age was 70y, 54% were male. The dominant mechanisms were OXA-48-like (n=4,884; 37.6%), NDM (n=3,927; 30.2%) and KPC (n=3,045; 23.4%); (Table 1) with large regional variation (Figure 1). NDMs became more dominant in 2023 than OXA-48-like. Screening samples accounted for 72% (n=9,378), and < 5% (n=591) were from invasive samples. Thirty-three patients with invasive samples had dual-mechanisms detected: 61% were NDM & OXA-48-like; *Klebsiella pneumoniae* was the dominant species (patients=21).

In 2023, the lowest reported resistance for the most frequently isolated species (n=5,535/5,711; Table 2) was for colistin (4-7%R) for all big-5 mechanisms in *Escherichia coli, Enterobacter* spp., *Citrobacter* spp., whilst higher for *Klebsiella* spp. and *Pseudomonas* spp. (10-17%R). Similarly, lower *Enterobacterales* resistance was observed for KPC and OXA-48-like to ceftazidime/avibactam (9-12%R) and amikacin (7-8%R), except for *Klebsiella* spp., which was higher (22%R). Ciprofloxacin and gentamicin had ≥ 35% resistance. Fosfomycin resistance in *E. coli* was low (≤ 9%).

Proportion of big-5 CPO isolates (n=5,535) resistant to other antimicrobials by most frequently isolates species in England, 2023

**Figure:**
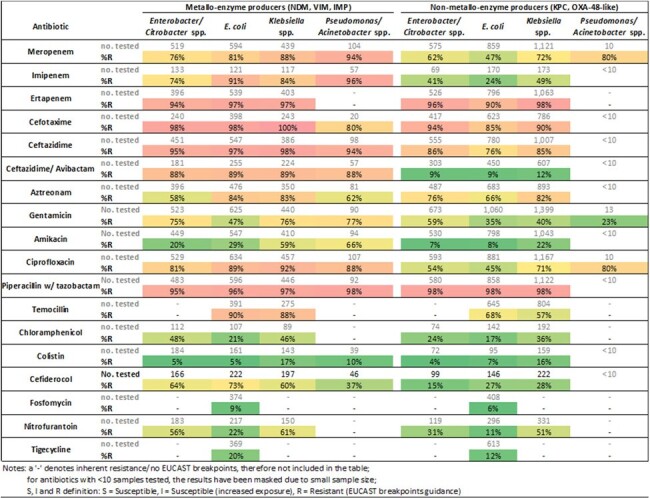

**Conclusion:**

Continued national CPO and associated AMR surveillance is crucial, particularly for new antimicrobials, to inform guidance and local, empiric prescribing by region, given the geographical variation in CPO mechanism. Further enhancement via data linkage to ethnicity and clinical characteristics to identify risk factors, and tracking changes in patients’ antibiograms over time would help further target national guidance.

**Disclosures:**

**All Authors**: No reported disclosures

